# Current perspectives on mass spectrometry-based immunopeptidomics: the computational angle to tumor antigen discovery

**DOI:** 10.1136/jitc-2023-007073

**Published:** 2023-10-29

**Authors:** Bing Zhang, Michal Bassani-Sternberg

**Affiliations:** 1 Lester and Sue Smith Breast Center, Baylor College of Medicine, Houston, Texas, USA; 2 Department of Molecular and Human Genetics, Baylor College of Medicine, Houston, Texas, USA; 3 Ludwig Institute for Cancer Research, University of Lausanne, Lausanne, Switzerland; 4 Department of Oncology, Centre Hospitalier Universitaire Vaudois, Lausanne, Switzerland; 5 Agora Cancer Research Centre, Lausanne, Switzerland

**Keywords:** Antigens, Neoplasm, Computational Biology, Immunity

## Abstract

Identification of tumor antigens presented by the human leucocyte antigen (HLA) molecules is essential for the design of effective and safe cancer immunotherapies that rely on T cell recognition and killing of tumor cells. Mass spectrometry (MS)-based immunopeptidomics enables high-throughput, direct identification of HLA-bound peptides from a variety of cell lines, tumor tissues, and healthy tissues. It involves immunoaffinity purification of HLA complexes followed by MS profiling of the extracted peptides using data-dependent acquisition, data-independent acquisition, or targeted approaches. By incorporating DNA, RNA, and ribosome sequencing data into immunopeptidomics data analysis, the proteogenomic approach provides a powerful means for identifying tumor antigens encoded within the canonical open reading frames of annotated coding genes and non-canonical tumor antigens derived from presumably non-coding regions of our genome. We discuss emerging computational challenges in immunopeptidomics data analysis and tumor antigen identification, highlighting key considerations in the proteogenomics-based approach, including accurate DNA, RNA and ribosomal sequencing data analysis, careful incorporation of predicted novel protein sequences into reference protein database, special quality control in MS data analysis due to the expanded and heterogeneous search space, cancer-specificity determination, and immunogenicity prediction. The advancements in technology and computation is continually enabling us to identify tumor antigens with higher sensitivity and accuracy, paving the way toward the development of more effective cancer immunotherapies.

## Introduction

**Figure 1:**
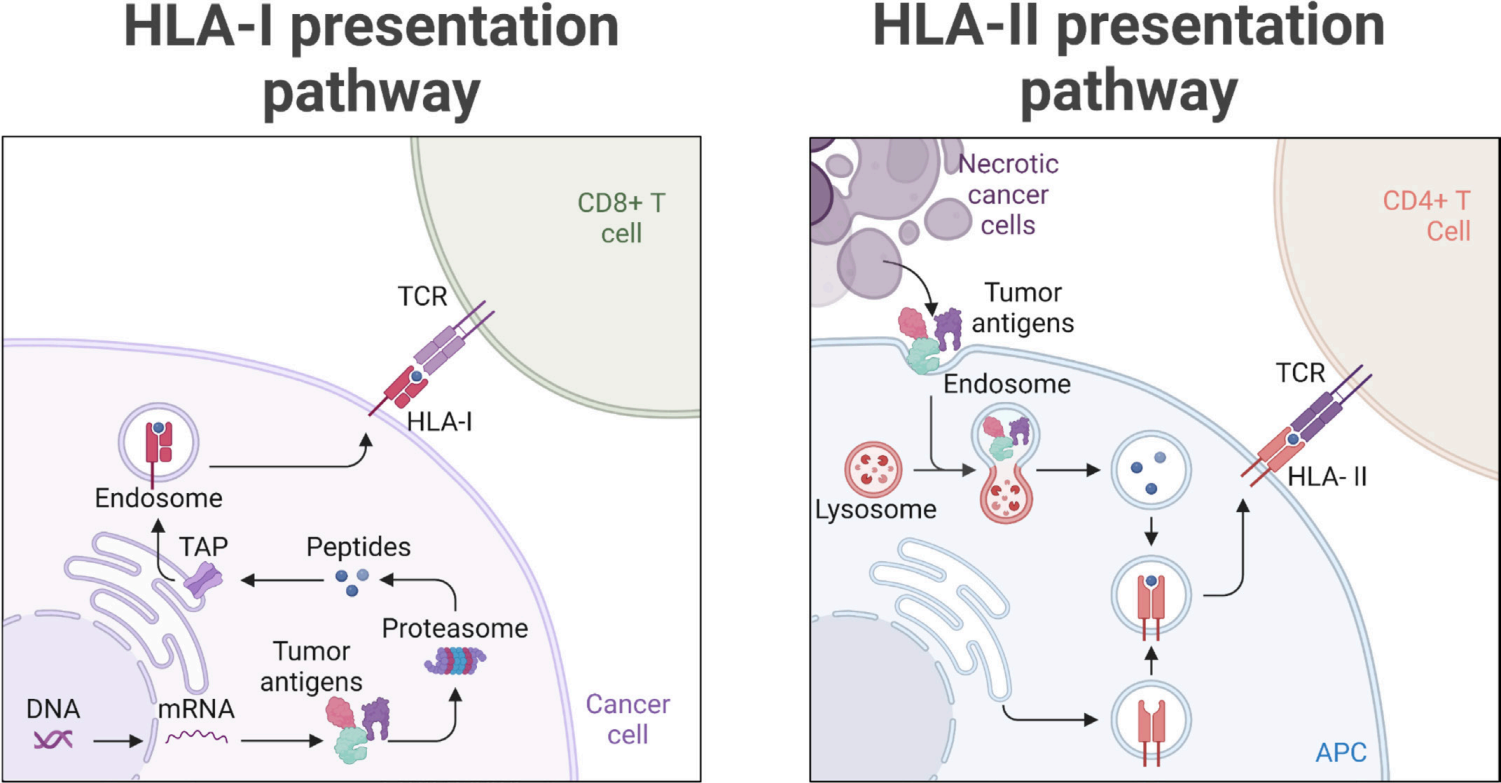
Schematic overview of the HLA-I and HLA-II presentation pathways enabling presentation of tumor antigens. APC, antigen presenting cell.

T cell-based recognition of tumor cells requires presentation of tumor antigens by the human leucocyte antigen (HLA) molecules. HLA class I (HLA-I) molecules that interact with CD8^+^ T cells present peptides derived mainly from proteasomal degradation of endogenous cytosolic proteins, while HLA class II (HLA-II) molecules expressed mainly on professional antigen presenting cells interact with CD4^+^ T cells and present peptides sampled from extracellular and intracellular proteins degraded via the endosomal pathway^1^ (figure 1). The repertoire of presented antigens, called the immunopeptidome, represents in real time the healthy state of cells. At the steady state, HLA-I and HLA-II immunopeptidomes consist of ‘normal’ self-peptides. Through the tumorigenic process, normal cells gradually accumulate genetic and other molecular alterations that lead to abnormal expression of mutated and other tumor-associated proteins, resulting in the presentation of tumor-specific and tumor-associated peptides, respectively, that can be specifically recognized as non-self by cytotoxic T cells through their T cell receptor, leading to T cell-mediated killing of cancer cells.^2^

**Figure 2:**
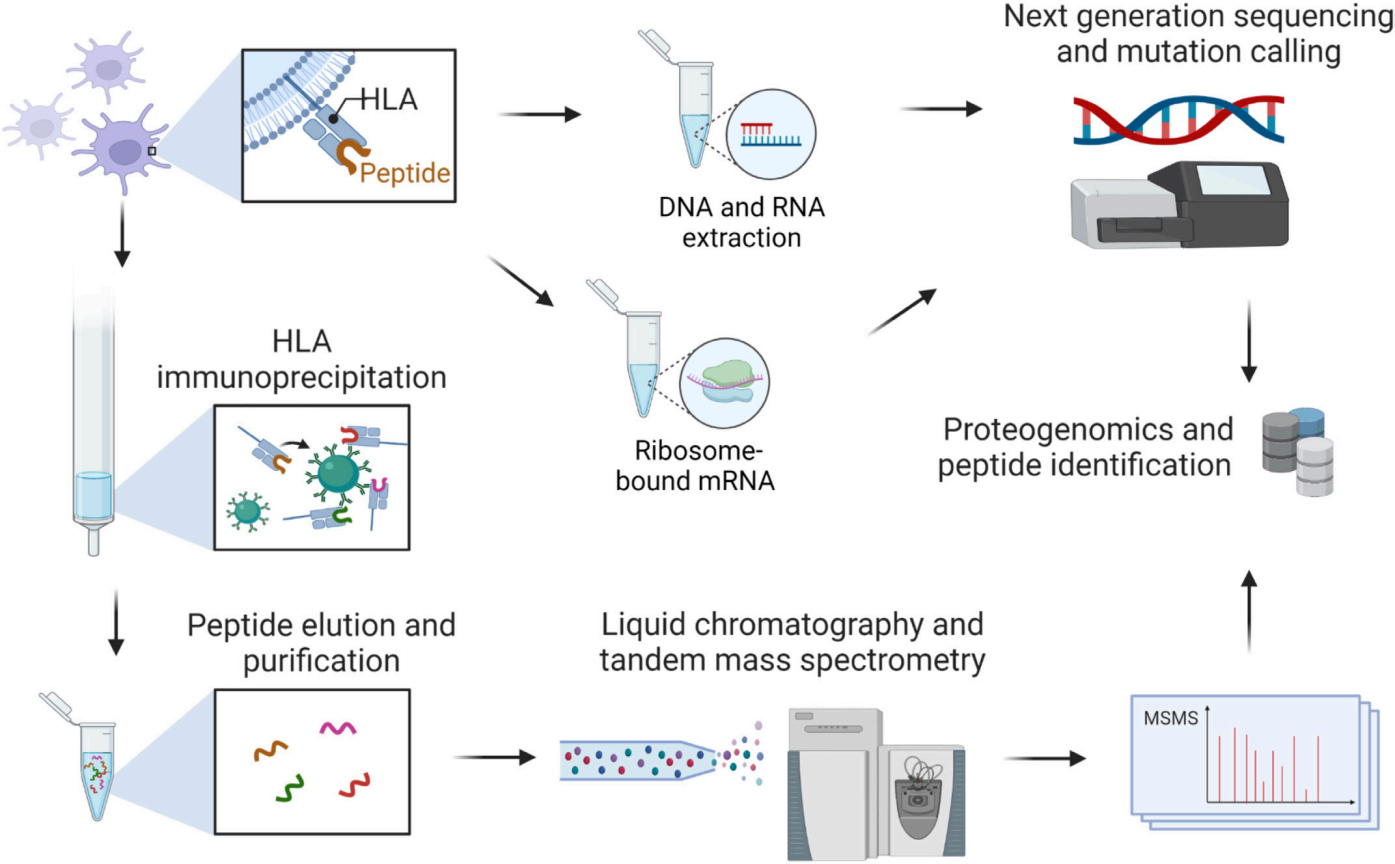
Antigen discovery with combining MS-based immunopeptidomics, genomics, transcriptomics and ribosomal footprinting. MS, mass spectrometry.

Cancer immunotherapies harness such natural anticancer immunity. Therefore, the identification of the particular immunogenic peptides that mediate spontaneous immune responses in patients with cancer, which can be unleashed by immune checkpoint blockade therapies or primed through vaccination, is of great importance.^3^ In recent years, immunopeptidomics, the application of mass spectrometry (MS) to identify HLA-bound peptides, coupled with novel experimental and computational proteogenomic approaches facilitated large-scale identification of various types of naturally presented tumor antigens^4–8^ (figure 2). The most common immunopeptidomics methodology is based on immunoaffinity purification of HLA complexes from detergent solubilized lysates, followed by purification and separation of the peptides by high-pressure liquid chromatography and their subsequent measurement by state-of-the-art sensitive MS instrumentation. The resulting MS data files are analyzed by computational algorithms, leading to the identification of thousands of peptides from tens of millions of cells or tens of mgs of tissues. Indeed, HLA peptide sources that are cancer-associated or cancer-specific can have a key role in cancer biology and in immune recognition.

Computational techniques are integral to the discovery of tumor antigens in immunopeptidomics. This review specifically examines the fundamental computational challenges in analyzing immunopeptidomics data and identifying tumor antigens. We focus on recent advancements in computational methods that enhance the sensitivity, reliability, and accuracy of HLA peptide and tumor antigen identification. Prior to delving into the computational aspects, we provide a concise introduction to the diverse sources of tumor antigens and the proteomics technologies employed in immunopeptidomics characterization. For in-depth information on these subjects, we refer readers to other recently published review articles.^3 9^ The primary objective of this review is to elucidate the critical role of computational approaches in immunopeptidomics-based tumor antigen discovery.

### Sources of tumor antigens

**Figure 3:**
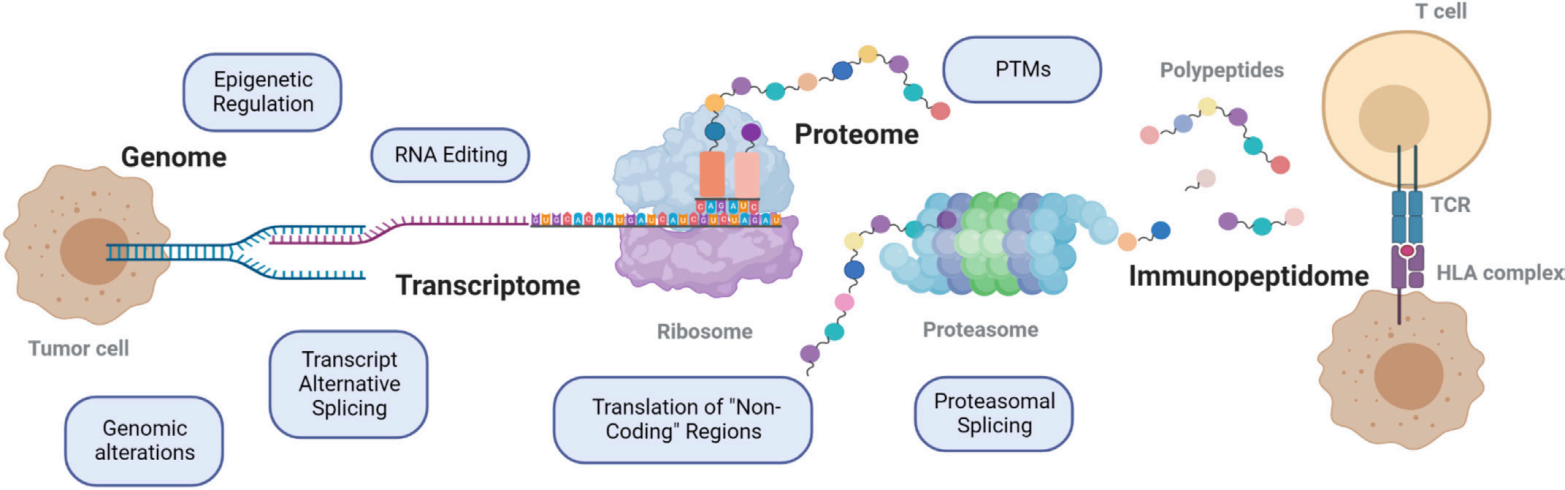
Various sources of tumor antigens. PTMs, post-translational modification.

Tumor antigens arise from various mechanisms (figure 3). HLA bound peptides that are encoded within the canonical open reading frames (ORFs) of coding genes are considered as canonical peptides and these have been widely explored. Canonical HLA bound peptides may result from post-translational events such as modifications, like phosphorylations.^10 11^ In addition, HLA bound peptides encoded in coding genes harboring somatic mutations, such as non-synonymous single-nucleotide variants (nsSNVs),^4^ nucleotide insertions or deletions (INDELs)^12^ and gene fusions,^13^ and alternatively spliced transcripts^14^ are also typically considered as canonical peptides if derived from canonical coding regions. In contrary, in recent years, proteogenomic-based immunopeptidomics studies demonstrated that HLA bound peptides can be derived from presumably non-coding regions of our genome (also called alternative, cryptic, or dark-matter), from alterations in the genome, epigenome, transcriptome, translatome, and the proteome. For example, post-transcriptional events, such as alternative splicing leading to intron retention, non-canonical translation initiation and codon read-through, as well as translation of long non-coding RNAs (lncRNAs), pseudogenes and transposablea elements (TEs) have been reported to generate non-canonical HLA peptides, some of which were demonstrated to be tumor-specific and immunogenic.^8 15 16^ Furthermore, proteasomal splicing^17^ and amino acid substitutions associated with deficiencies in translation^18^ have been proposed as additional sources of HLA ligands. It is expected that once the existence of any of the above non-canonical sources will become more evident, common and thoroughly validated, they will gradually be considered and annotated as canonical.

### Proteomics technologies used in immunopeptidomics characterization

Often, the collective identification and quantification of purified HLA peptides by MS is discovery oriented.^19 20^ Data-dependent acquisition (DDA) MS approaches are commonly used because they generate high-quality references of peptide tandem MS/MS fingerprints. Precursors for fragmentation in a DDA measurement are selected based on various factors, such as ion intensity and charge state, and therefore, DDA acquisition is ideal for confident identification, for example, when post-translational modifications (PTMs) or non-canonical sources are explored. While DDA methods often have low reproducibility between samples, labeling approaches overcome issues of low abundance samples and the resulting low quality of MS/MS spectra. For example, with tandem mass tag, individual samples are barcoded with an array of isobaric tags and combined for a single MS measurement. In immunopeptidomics, it has been shown to improve detection coverage and the identification of low abundant peptides.^21^ Recently, a new approach demonstrated usage of recombinant heavy-isotope-coded peptide major histocompatibility complexes (hipMHCs) as internal standards for normalization correction to enhance reproducibility of immunopeptidomics measurements. hipMHCs are added to the samples at the beginning of the processing workflow, and are purified together with the endogenous complexes, hence, enabling accurate comparisons between different experimental conditions in both label-free and multiplexed labeled immunopeptidomics analyses.^19^

In general, data-independent acquisition (DIA) is more suitable for comparative or differential immunopeptidomics. In DIA, all precursor ions are isolated and fragmented in an unbiased manner within shifted and overlapping isolation windows, therefore, peptide reproducibility and quantification across multiple samples are greatly enhanced. Several immunopeptidomics studies optimized DIA acquisition parameters and the computation approach for peptide identification that required spectral libraries.^19 22–25^ This approach limits the discovery of novel or non-canonical peptides. Library-free approaches for DIA data analyses, and hybrid approaches that combine both spectral library and database search, have been developed and are used for proteomics studies. These will likely be adopted soon by the immunopeptidomics community as well to improve quantitative precision and increase the number of quantified HLA bound peptides.^26 27^

The most robust and accurate method to quantify a defined set of ions in complex peptide mixtures is by targeted MS approaches such as parallel reaction monitoring and selected reaction monitoring. Combined with spik-in of synthetic isotopically labeled counterpart peptides, these methods can validate the correct identification of the endogenous peptides which is a critical step for determining the authenticity of novel and unexpected non-canonical peptides.^8^ Targeted MS methods can quantify the abundance and copy number of specific HLA bound peptides on cell surfaces over time. For example, Croft *et al* 28 quantified the presentation of eight vaccinia virus MHC-I peptides on infected cells. It is important to note that they found a complete disconnect between the peptides’ abundance and their immunodominance. Therefore, even in the case of non-self-peptides from pathogens, one should not assume that peptide abundance is directly associated with its recognition by T cells.

### Computational analysis of untargeted immunopeptidomics data

A typical untargeted immunopeptidomics experiment may generate hundreds of thousands of MS/MS spectra, which need to be analyzed by computational tools to identify peptides presented by HLA molecules. Commonly used methods for peptide identification include database searching, spectral library searching, and de novo sequencing.^29^ Database searching involves comparing the experimentally acquired MS/MS spectra against theoretical spectra derived from in silico digestion of a reference protein database, such as Ensembl, Refseq, or UniProt. Spectral library searching is similar to database searching, but instead of searching against a reference protein database, the method searches against a reference library of previously identified spectra. De novo sequencing involves predicting the sequence of peptides directly from the MS data without the use of a reference database or library. False discovery control is critical in peptide identification from MS data. By adding incorrect, ‘decoy’ sequences or spectra to the search space, the target-decoy approach provides a simple but powerful method for false discovery rate (FDR) estimation in database searching^30^ and spectral library searching.^31^ Effective control of FDR remains challenging in de novo sequencing.

**Figure 4:**
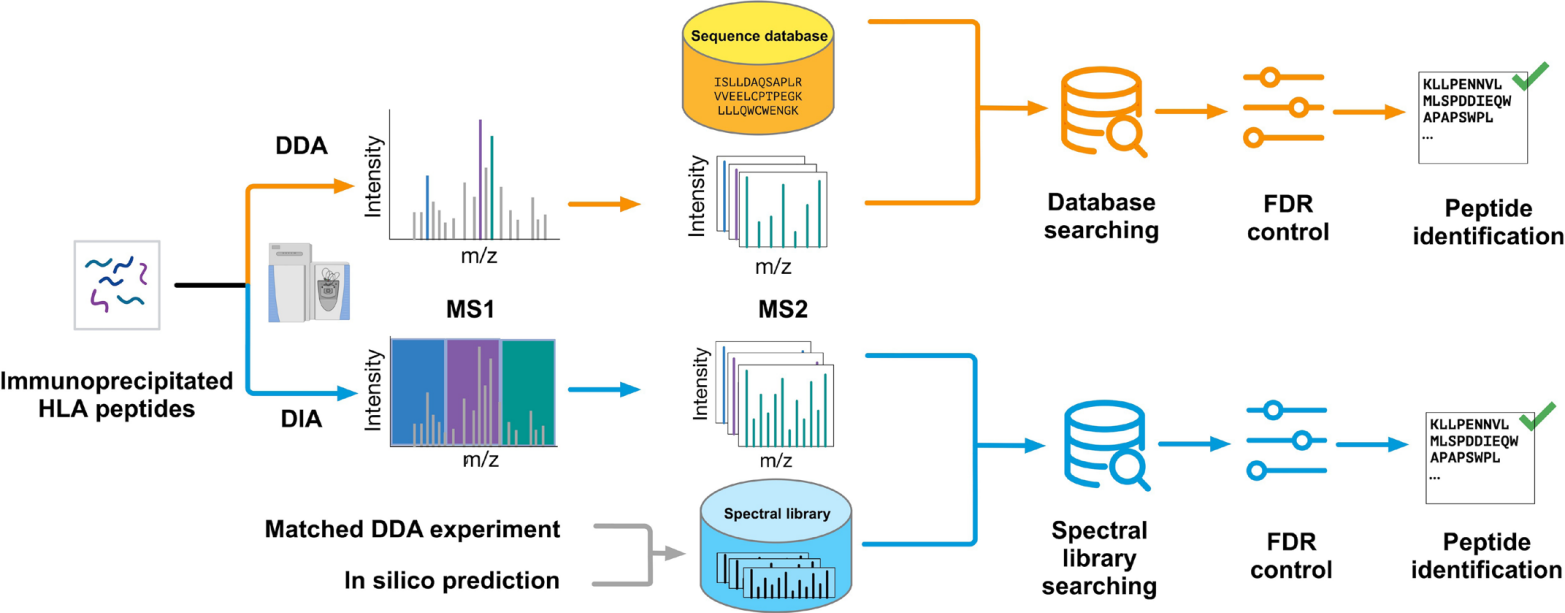
Typical workflows for the analysis of DDA and DIA immunopeptidomic data. DDA, data-dependent acquisition; DIA, data-independent acquisition; FDR, false discovery rate; HLA, human leucocyte antigen.

In DDA immunopeptidomic data analysis, database searching is the most widely used method (figure 4). Database searching tools, such as Comet,^32^ MS-GF+,^33^ X!Tandem,^34^ MaxQuant,^35^ and Mascot,^35 36^ can be used for such analysis. These search engines can only include a small number of prespecified PTMs in database searching, referred to as closed search. The more recently developed open search engines, such as MSFragger^37^ and open-pFind,^38^ allow unbiased identification of all PTMs on HLA-bound peptides from non-PTM-enriched samples.^11 39^ It has been shown that the choice of search engine has a significant impact on the number of peptides that can be confidently identified from the same DDA experiment,^39 40^ and the overlap among peptides identified by different search engines is moderate.^41^ This may suggest inferior sensitivity of these search engines, which are originally developed for the analysis of shotgun proteomics data.

In shotgun proteomics, proteins are digested into peptides by trypsin or other enzymes before LC-MS/MS analysis,^42^ and the sequence specificity of enzyme cleavage enables an enzyme-specific search within a constrained database search space. Because immunopeptidomic experiments do not require enzymatic digestion, a non-enzyme-specific search in a much larger search space leads to lower sensitivity in peptide identification.^43^ Several computational methods have been developed to address this challenge. Based on the assumption that immunopeptidomes contain a limited number of recurring peptide motifs corresponding to HLA specificities, MS-Rescue learns sequence motifs based on peptides identified from high-scoring peptide-spectrum matches (PSMs) and then uses the learned information to rescue PSMs with relatively lower scores but a high motif score.^44^ Using a semisupervised machine learning model implemented in Percolator,^45^ MHCquant rescores Comet identified PSMs by incorporating features not initially used in PSM scoring.^46^ With the advancements of deep learning in proteomics,^47^ it is now possible to accurately predict many peptide features, such as retention time and fragment ion intensity using deep learning tools such as Prosit,^48^ AutoRT,^49^ DeepMass^50^ and pDeep.^51^ Incorporating deep learning derived features in Percolator-based PSM rescoring has been shown to significantly improve peptide identification in the analysis of DDA immunopeptidomics data.^41 52^

In DIA experiments, because all precursor ions within an isolation window are fragmented together, the highly complex fragment ion mass spectra complicate peptide identification. Although methods have been developed to first deconvolute the complex MS/MS spectra and then perform database searching, spectral library searching is a preferred method in DIA data analysis (figure 4). Tools for library-based DIA data analysis include OpenSWATH,^53^ Spectronaut,^54^ Skyline,^55^ DIA-NN,^56^ EncyclopeDIA,^57^ MaxDIA,^58^ PEAKS^59^ among others. Some of these tools can also be run in a library-free mode. Due to its user-friendly features, Spectronaut is a popular choice in DIA data analysis. More recent tools such as DIA-NN leverages deep learning to improve peptide identification. Several benchmarking studies have been performed to evaluate DIA data analysis pipelines in the context of proteomics and phosphoproteomics.^60–63^ In the most recent study using the latest versions of DIA-NN, Spectronaut, MaxDIA and Skyline, DIA-NN is recommended for global DIA proteomic data analysis given the overall superior performance and the open-access feature, whereas complementary performance of DIA-NN and Spectronaut is reported in phosphoproteomic data analysis.^63^ For immunopeptidomic data analysis, a recent benchmarking study comparing DIA-NN, PEAKS, Skyline and Spectronaut shows that PEAKS and DIA-NN provides higher sensitivity and reproducibility whereas Skyline and Spectronaut provides higher specificity, and the combination of multiple tools provides the greatest coverage while a consensus approach leads to the highest accuracy.^64^

In addition to software selection, the choice of spectral libraries is also an important consideration in library-based DIA data analysis. Experimental libraries constructed from DDA analysis of the same or similar samples under comparable LC-MS/MS settings are routinely used in DIA data analysis. However, this approach is time-consuming, consumes more materials, and limits the identification by DIA to the peptides identified by DDA. In silico libraries created through deep learning tools that predict fragment ion intensity and retention time for peptide sequences address these limitations and have been shown to achieve similar or better performance in DIA data analysis.^65^ This is particularly attractive in the immunopeptidomic analysis of small and precious clinical samples such as tumor tissue biopsies. Efforts have been made to benchmark DIA analysis tools and their combinations with library construction methods based on tryptic MS data,^61 63^ similar benchmarking analysis based on immunopeptidomic data would be very helpful.

### Identification of tumor antigens

**Figure 5:**
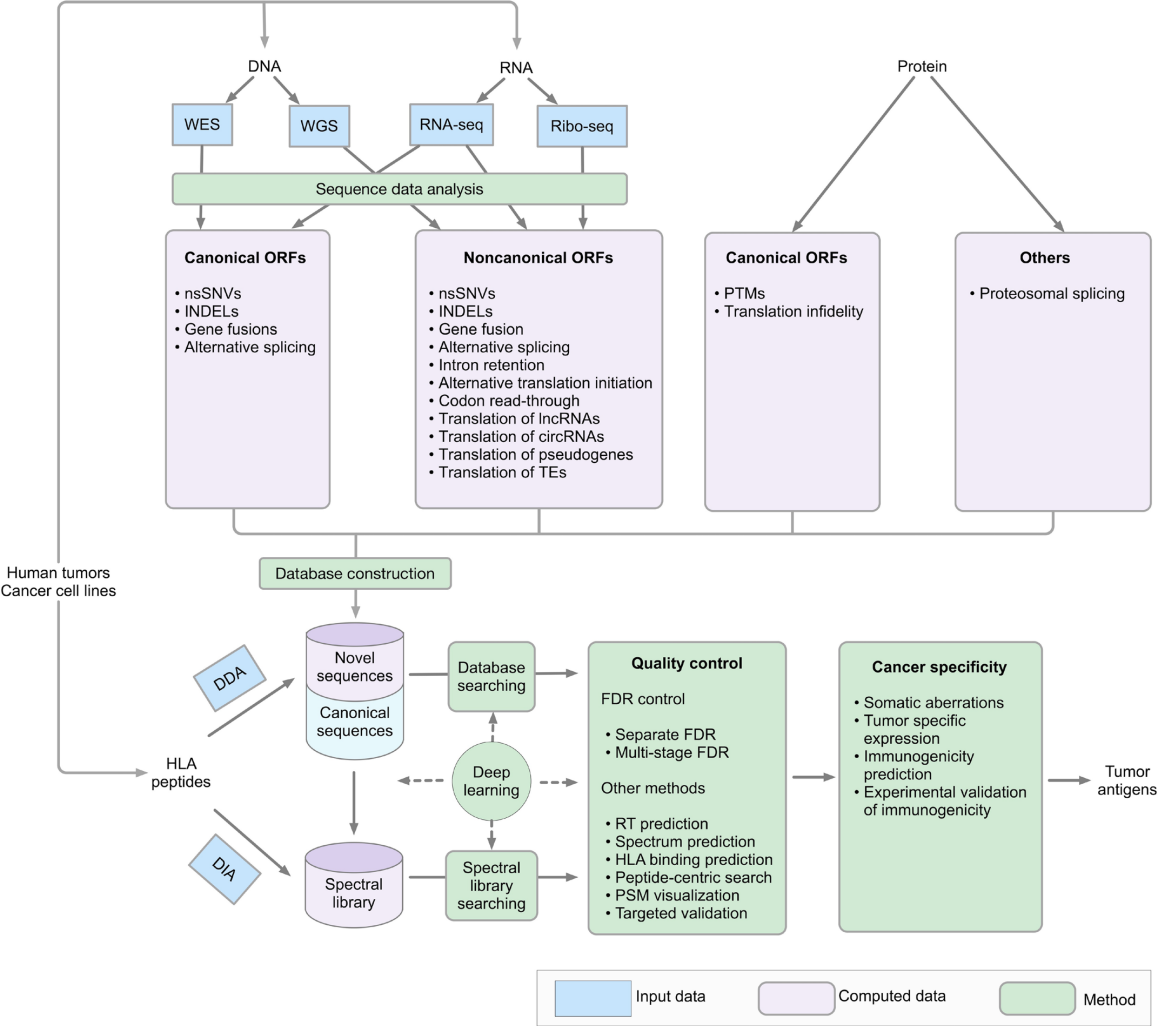
Schematic overview of the tumor antigen identification workflow. DDA, data-dependent acquisition; DIA, data-independent acquisition; FDR, false discovery rate; HLA, human leucocyte antigen; INDELs, nucleotide insertions or deletions; nnSNVs, non-synonymous single-nucleotide variants; ORFs, open reading frames; PSMs, peptide-spectrum matches; PTMs, post-translational modifications; TEs, transposable elements; WES, whole exome sequencing; WGS, whole genome sequencing.

Novel protein sequences resulting from cancer-specific aberrations at genomic, transcriptomic, and translational levels are promising sources of tumor antigens. The proteogenomics approach^66^ that incorporates DNA sequencing, including whole exome sequencing (WES) and whole genome sequencing (WGS), RNA sequencing (RNA-seq), and ribosome sequencing (Ribo-seq) data into MS-based proteomics and immunopeptidomics data analysis provides a powerful means for identifying tumor antigens. This approach has been widely used in database searching-based analysis of DDA immunopeptidomics data by generating customized protein databases that extend the reference protein database to include novel protein sequences predicted based on WES, WGS, RNA-seq, or Ribo-seq data.^3^ Recently, codon reassignment during translation has also been reported as a source of neoantigens, which can also be identified through searching immunopeptidomics data against customized protein databases including novel protein sequences derived from the codon reassignment of interest.^18^ For DIA data analysis, RT and fragment ion intensity can be predicted for sequences in the customized protein databases using deep learning tools, and the predicted RT and MS/MS spectra can be used for the identification of both canonical and non-canonical peptides.^22^ There are several key considerations in the proteogenomics-based approach, including accurate DNA, RNA and ribosomal sequencing data analysis, carefully designed plans for incorporating predicted novel protein sequences into reference protein database, special quality control in MS data analysis due to the expanded and heterogeneous search space, cancer-specificity determination for the identified HLA peptides, and immunogenicity prediction. Figure 5 provides a schematic overview of the tumor antigen identification workflow, and related computational tools are summarized in online supplemental table 1.

10.1136/jitc-2023-007073.supp1 Supplementary data

### Analysis of DNA, RNA, and ribosome sequencing data

WES data are the most frequently used for the identification of coding DNA sequence variants such as nsSNVs and INDELs. In a benchmarking study evaluating the performance of four popular short read aligners (Bowtie2,^67^ BWA,^68^ Isaac,^69^ and Novoalign) and nine variant calling and filtering methods (Clair3,^70^ DeepVariant,^71^ Octopus,^72^ GATK,^73^ FreeBayes,^74^ and Strelka2^75^) using 14 ‘gold standard’ WES and WGS datasets, DeepVariant consistently showed the best performance and the highest robustness.^76^ Analysis of WES data from tumor and matched germ line (eg, blood) samples enable the identification of somatic variants, which are the sources of the traditionally considered tumor-specific neoantigens. Many computational tools have been developed for somatic mutation calling from WES data,^77–83^ and a systematic benchmarking study from the ICGC-TCGA DREAM Somatic Mutation Calling Challenge showed that an ensemble of computational pipelines always outperforms the best individual pipeline with regard to both sensitivity and specificity.^84^ WES prioritizes the coverage of annotated coding genes. If non-canonical coding regions predicted from RNAseq or ribosomal data (see below) are of interest, using WGS data for somatic mutation calling can provide better coverage of these regions.^15^

RNASeq data provides comprehensive information on nucleotide variation and transcript identity and abundance, both are useful for sample specific customized database construction.^85^ RNA-level SNVs reflect not only DNA variations but also RNA-editing events. Driven by a post-transcriptional regulatory process, RNA editing derived peptides can be presented by HLA and elicit immune responses.^86^ General transcript assembly tools such as Cufflinks^87^ and StringTie^88^ report both annotated and novel transcripts from RNA-seq data. Specialized computational tools have also been developed to identify specific types of aberrantly expressed transcripts. Gene fusion is an important source of neoantigens.^89^ Fusion RNAs may arise from chromosomal rearrangements or aberrant RNA splicing, and both can be identified from RNA-seq data. A study benchmarking 23 tools for fusion prediction using simulated and real RNA-seq data identified STAR-Fusion,^90^ Arriba,^91^ and STAR-SEQR^90^ as the fastest and most accurate for fusion detection on cancer transcriptomes.^90^ Intron retention is another source of neoantigens in cancer^92^ and can be detected from mRNA-seq data using tools such as IRFinder.^93^ Due to frequent global loss of DNA methylation in human cancers, aberrant expression of transcripts derived from endogenous TEs represents another source of tumor antigens.^94 95^ Accurate identification and quantification of TE-derived transcripts in short-read RNA-seq data can be challenging due to the repetitive nature of their sequences. REdiscoverTE^94^ has been developed to address this challenge, and long-read RNA-seq may enable more accurate analysis of expressed TEs. Circular RNAs resulting from back-splicing events during pre-mRNA splicing can be identified and quantified by CIRIquant.^96^ CircRNAs are frequently dysregulated in cancer cells.^97^ Although lacking a 5′ cap, they can be translated using cap-independent mechanisms,^98^ raising their potential as a source of tumor antigens.

Ribo-seq provides experimental information on the actively translated regions of the genome, revealing the existence of thousands of ORFs within long non-coding RNAs (lncRNAs) and regions of protein-coding genes that were previously thought to be untranslated (UTRs). Translated sequences identified by Ribo-seq that have not already been annotated by reference annotation projects are known as Ribo-seq ORF, non-canonical ORF, alternative ORF, novel ORF, or when less than 100 amino acids in size, small ORF or short ORF.^99^ Ribo-seq ORFs are infrequently identified in shotgun proteomics data, possibly due to unstable protein products. Interestingly, in an effort to identify proteomic evidence from PeptideAtlas for Ribo-seq ORFs, the majority of observed peptide evidence was found in immunopeptidomics datasets,^99^ suggesting unstable source proteins could serve as a source of HLA peptides. Indeed, searching immunopeptidomics data against generic or sample-specific Ribo-seq inferred reference protein databases enabled the identification of many HLA-I bound peptides.^8 15^ The major computational challenge in detecting translation using Ribo-seq data is the discrimination of the signal obtained with genuine ribosome footprints from mapping artifacts and other RNA fragments. Computational tools have been developed to address this challenge using different approaches.^100^ For example, ribotricer detects actively translating ORFs by directly leveraging the three-nucleotide periodicity of Ribo-seq data.^101^ RiboHMM uses a hidden Markov model,^102^ RibORF uses a Support Vector Machine classifier,^103^ and PRICE uses an EM algorithm^104^ to detect translating ORFs. Ribo_TISH is able to use Ribo-seq data enriched at starts of initiation in addition to regular Ribo-seq data.^105^ Predictions from different computational tools may differ considerably, and it is not easy to benchmark these tools because of the lack of gold standard sets of translated ORFs. A recent community-led effort has produced a standardized catalog of 7264 human Ribo-seq ORFs,^99^ which provides a unified resource to facilitate Ribo-seq research and will benefit the integration of non-canonical ORFs into immunopeptidomics data analysis.

### Incorporating predicted sequences into reference protein database

Novel peptide sequences resulting from nsSNVs and in-frame INDELs can be generated by replacing the affected amino acids in the canonical reference protein sequence. DNA sequencing is better suited for calling somatic mutations than RNA-Seq, but their combination can help prioritize somatic mutations that are expressed at the RNA level, which are required for protein production. Many studies include only somatic mutations in novel peptide sequence generation; however, neglecting nearby germline variants may result in missed opportunities for identifying potential neoantigens.^106^ How to handle nsSNV combinations in customized database generation and MS data analysis remains an open question. Comet has been extended to automatically analyze global amino acid variants encoded in the PSI extended FASTA format,^107^ but this feature has rarely been used in immunopeptidomics studies. In addition to nsSNVs, codon reassignment during translation or translational infidelity may also lead to novel peptide sequences.^18 108^ In this case, the translational alterations of interest could be introduced globally during reference protein database construction, but the canonical sequences should also be kept in the database to avoid false positive identifications caused by the lack of competition from canonical sequences^109^ Out-of-frame INDELs cause frameshifts to coding sequence, which can lead to novel protein sequences. Of note, frameshift mutations frequently lead to premature termination codon (PTC), and PTC-bearing transcripts are often degraded by nonsense-mediated decay (NMD). Therefore, integration of matched RNA-seq data would be useful to identify PTC-bearing transcripts escaping NMD, which is a promising source of neoantigens.^110 111^

To generate protein databases from RNA-seq data, assembled transcripts can be in silico translated into amino acid sequences. For stranded RNA-seq data, which provides information about the directionality of the transcripts, a three-frame translation is performed. A six-frame translation is required for unstranded RNA-seq data, which lacks information about the directionality of the transcripts. These processes vastly increase the database size. To reduce database size, transcript abundance could be used to filter out lowly expressed transcripts that are unlikely to produce detectable HLA peptides.

Ribo-seq data provide information about the correct coding frame for each transcript and are well suited for the de novo reference protein database construction. Some ribosome profiling methods focus on translation initiation and enrich ribosomes at the start of translation initiation for analysis.^105^ In this case, localization of start codons identified from such experiments can be integrated with de novo assembled transcripts to generate customized protein databases.^112^

Computational tools and workflows have been developed to facilitate customized database construction, such as CustomizedProDB,^113^ JUMPg,^114^ PROTEOFORMER,^115^ and pgdb.^116^

### Tumor antigens generated from post-translational processes

Post-translational processes such as PTMs and proteosomal splicing further expand the landscape of tumor antigens. Comprehensive identification of modified peptides from non-PTM enriched immunopeptidomics experiments requires the use of open search engines. Systematic application of open-pFind to 43 published human immunopeptidomic datasets identified 55 710 modified HLA class I peptides and 92 203 modified HLA class II peptides.^39^ Similarly, applying the MSFragger-based Protein Modification Integrated Search Engine to HLA I immunopeptidomics data from 210 samples identified thousands of modified HLA class I peptides.^11^ To characterize a specific type of modified peptides, PTM-specific peptide enrichment, such as enrichment of phosphorylated peptides with immobilized metal affinity chromatography, can be used.^117^

Proteasomal spliced peptides (PSPs), generated by the proteasome through the splicing of two distinct peptide fragments, were first reported by Hanada et al in 2004.^118^ PSPs have been shown to be presented on HLA molecules and to induce antigen-specific T cell responses in a melanoma patient^119^ and hence their large-scale identification through MS has become an active area of research. However, there are several important challenges associated with MS-based identification of PSPs. PSPs can be generated from all possible combinations of peptide fragments resulting in an enormous space search. Database size inflation subsequently compromises FDR calculations leading to propagation of false identifications.^17^ Indeed, first studies reported that PSPs comprise 30%–40% of the immunopeptidomes,^120 121^ yet following reanalysis of these datasets, incorporating de novo sequencing and researching techniques estimated an upper bound values of around 3%.^122^ A dedicated search program called Neo-Fusion, was created for discovering spliced peptides in tandem MS data,^123^ by using two separated ion database searches to identify the two halves of each spliced peptide, and then to infer the full spliced sequence. With this tool, a recent study independently reported again the identification of potential PSPs that represented less than 3.1% of the total canonical peptidome.^124^

### Special quality control in non-canonical peptide identification

One challenge in proteogenomics-based identification of non-canonical HLA-bound peptides from immunopeptidomics data is accurate FDR control. This challenge is illustrated above for PSPs, but it is common for other types of non-canonical peptides. In general, predicted non-canonical proteins are less likely to produce HLA-bound peptides than canonical proteins, and different types of predictions also come with different levels of confidence. For example, predictions based on Ribo-seq data are more reliable than those based on RNA-seq data. Accordingly, direct application of the target-decoy strategy without discriminating canonical and different types of non-canonical peptides would result in an underestimate of the true FDR for non-canonical peptides, thereby raising the possibility of false-positive non-canonical peptide identifications.

To address this limitation, two alternative methods for estimating FDR have been developed: the separate FDR method and the multistage FDR method. The separate FDR method calculates FDRs for canonical and different types of non-canonical peptides separately, whereas the multistage FDR method requires multiple stages of analysis. In the first stage, MS/MS data are matched against a database with canonical proteins, and confidently identified spectra are removed. Each following stage involves matching the remaining spectra against the group of non-canonical proteins with the highest confidence and calculating the FDR based on the search results. For both approaches, when the number of identifiable non-canonical peptides is small, FDR estimation may be inaccurate. The multistage FDR is further vulnerable to false negatives because an MS/MS spectrum generated from a non-canonical peptide may be incorrectly matched to a canonical peptide in the first stage and excluded from the downstream analysis.

Due to the challenges in accurate FDR control, additional validation steps could be taken to further assess or reduce errors. First, machine learning and especially deep learning models enable accurate prediction of many peptide features such as retention time, fragment ion intensity, and HLA binding affinity.^48–51 125–127^ If these predictions are not already used in the step of peptide identification, they can provide independent assessment of the novel peptide identifications. Second, traditional database searching methods consider only a small number of protein modifications due to search complexity, and the target-decoy based FDR estimation lacks rigorous quality control for individual PSMs. False positives can occur when a spectrum matched to a novel peptide is actually derived from a canonical peptide containing a chemical or PTM not accounted for in the database searching. This problem can be potentially addressed by a peptide-centric analysis. By shifting the focus from interpreting all observed MS/MS spectra in a study to validating a small number of candidate novel peptide identifications, this approach provides statistical assessments for individual PSMs and also enables comprehensive examination of peptide modifications to reduce false discoveries. Originally demonstrated in PepQuery^128^ for tryptic proteomic data analysis, this approach has also been modified for the analysis of immunopeptidomics data.^49^ In addition to these computational methods, quality assessment can also be achieved by manual examination of the PSMs using visualization tools such as PDV.^129^ Finally, targeted proteomic analysis with spiked-in heavy-isotope labeled peptides can provide ultimate experimental validation of the selected novel peptides.

### Cancer-specificity determination

Cancer specificity and immunogenicity are key requirements of clinically actionable tumor antigens. Neoantigens resulting from somatic mutations are the most confident group of cancer specific antigens because cancer specificity is determined during somatic mutation calling in which tumor sequences are directly compared with germline sequences. However, most somatic mutation derived neoantigens are patient specific, limiting their potential application as targets of prefabricated vaccines or T cell products.

Cancer specificity of non-canonical antigens resulting from transcriptional, translational, and post-translational aberrations are more difficult to determine. One approach is to perform parallel omics analysis on tissue-matched normal samples to assess cancer-specificity of the non-canonical proteins predicted by RNA-seq or Ribo-seq data or cancer-specificity of non-canonical epitopes identified from immunopeptidomics data. Elimination of non-canonical proteins predicted by RNA-seq or Ribo-seq can be performed by removing them from the customized databases used for immunopeptidomics data analysis or by removing non-canonical epitopes that are mapped to proteins with expression evidence in normal samples. The former approach may significantly reduce the search space in immunopeptidomics data analysis, but it could potentially lead to false positive cancer-specific peptide identifications because the spectra supporting a cancer-specific peptide identification may have better match to another peptide that are expressed in both tumor and normal samples. Subtraction of non-canonical epitopes that are not cancer specific may also leverage public databases and the analysis may be extended to include all non-immune privileged tissues. The TCGA^130^ and CPTAC^131 132^ datasets can be used to assess differential abundance of non-canonical proteins at RNA and protein levels across many cancer types. Gene expression of the source genes of non-canonical epitopes across different healthy tissues can be further investigated using the GTEx datasets.^133^ Moreover, immunopeptidomics data generated from non-cancerous samples, such as those from the HLA Ligand Atlas^134^ and the caAtlas,^39^ provide comprehensive references for assessing tumor specificity of non-canonical epitopes.

Immunogenicity in human subjects is an important determination of cancer specificity. Computational prediction of immunogenic peptides has been an active research area, and multiple computational models have been developed during the past decades. A recent benchmarking study^135^ evaluating seven publicly available models shows that none of them perform substantially better than random or offer clear improvement beyond HLA ligand prediction for predicting immunogenic peptides from an emerging virus such as severe acute respiratory syndrome coronavirus 2. For identifying immunogenic neoantigens, several models, including Gao *et al,* 136 NetTepi,^137^ PRIME,^138^ and the eluted ligand (netMHCpan_EL) and binding affinity (netMHCpan_BA) predictions from NetMHCpan 4.0^125^ performed better than random, but all with suboptimal performance scores, suggesting considerable room for improvement. Immunogenicity of the prioritized tumor antigens can be further experimentally evaluated using IFN-gamma ELISpot assay or other approaches.

### Concluding remarks and future directions

The field of cancer immunopeptidomics is rapidly evolving due to experimental and computational advancements, as well as its integration with cancer-specific aberrations identified from DNA, RNA, and ribosome sequencing data. While early studies were focused on neoantigens derived from somatic mutations, recent research has emphasized the importance of non-canonical antigens as a broader source of tumor antigens. Consequently, our understanding of naturally presented tumor antigens has expanded significantly, presenting new prospects for cancer immunotherapy.

Despite exciting advancements, sensitive and accurate identification of tumor antigens from immunopeptidomics data remain challenging. Indeed, most of the MS/MS spectra generated in immunopeptidomics experiments cannot be mapped to peptides based on the existing algorithms. Proteogenomics-based novel peptide sequence identification can benefit from new DNA, RNA, and ribosome sequence data analysis algorithms. Even for the most extensively studied topics such as variant calling from WES data, significant improvements are still being continuously made through new algorithms such as DeepVariant.^76^ These new advancements should be incorporated into immunopeptidomics data analysis pipelines. Moreover, due to intratumor heterogeneity, leveraging single cell RNA-seq data may enable identification of tumor antigen source genes expressed in a subset of cells and their inclusion in customized databases to allow eventual detection in the immunopeptidome.^8^ New proteomics data analysis algorithms can also improve MS/MS spectra identification rate. It has been shown that many MS/MS spectra are chimeric spectra, and algorithms such as CHIMERYS^139^ could be used to support interpretation of such spectra. De novo peptide sequencing also holds great potential in discovering novel peptide sequences. A new platform integrating deep learning-based solutions of spectral library search, database search, and de novo sequencing has been shown to boost sensitivity on both DDA and DIA immunopeptidomics data.^140^ To facilitate new method development, it is critical to make immunopeptidomics data publicly available and follow the FAIR principle^141^ in data sharing. Meanwhile, it is equally important to make computational pipelines used in published studies available. Because computational pipelines for tumor antigen discovery usually involve many components, it is useful to dockerize individual analytical components and implement the pipeline using workflow languages to improve reproducibility and reusability. Several databases, such as SysteMHC Atlas,^142^ HLA Ligand Atlas,^134^ and caAtlas^39^ have made antigens identified from a large amount of immunopeptidomics data on healthy or cancer samples easily available to the public through dedicated web portals. Combining these resources into a unified platform would be highly beneficial.

One major obstacle to the clinical translation of immunopeptidomics is the limited availability of clinical materials. Advanced proteomics technologies, such as ion mobility separation-based timsTOF MS, have the potential to detect HLA-presented peptides with higher sensitivity, which is critical when the available material is limited, as in core needle biopsies. Moreover, to enable multiomics analysis based on small clinical samples, it is crucial to develop standardized sample preparation protocols to enable such analysis. Close collaboration among experimentalists, computational biologists, oncologists, and clinicians is essential to realizing the clinical potential of tumor antigens identified from immunopeptidomics. By working together, we can overcome the challenges of clinical translation and advance the field toward personalized cancer immunotherapy.
