# Supplementary material for: Current perspectives on mass spectrometry-based immunopeptidomics: the computational angle to tumor antigen discovery
Source: J Immunother Cancer. 2023 Oct 29;11(10):e007073. doi: 10.1136/jitc-2023-007073 (PMC10619091; doi:10.1136/jitc-2023-007073)
Supplement: Supplementary data [file jitc-2023-007073supp001.pdf]

Table 1. A non-exhaustive list of computational tools and resources relevant to tumor antigen identification

Name	URL	Ref	Remarks
WES/WGS data analysis			
Bowtie2	https://bowtie-bio.sourceforge.net/bowtie2/index.shtml	[67]	A widely used short read aligner for aligning DNA sequencing reads to reference genomes.
BWA	https://bio-bwa.sourceforge.net/	[68]	A gold standard solution for short read alignment in medical genetics.
Isaac	https://github.com/sequencing/isaac_aligner	[69]	A widely used short read aligner known for its speed in mapping DNA sequencing reads.
Novoalign	https://www.novocraft.com/products/novoalign/		A commercial read alignment package known for its accuracy in mapping DNA sequencing reads.
Clair3	https://github.com/HKU-BAL/Clair3	[70]	A germline small variant caller using pileup data and deep neural networks.
DeepVariant	https://github.com/google/deepvariant	[71]	A SNP and small-indel variant caller using deep neural networks.
Octopus	https://github.com/luntergroup/octopus	[72]	A unified haplotype-based method for variant calling.
GATK	https://gatk.broadinstitute.org/hc/en-us#info-tab	[73]	A comprehensive genomic analysis toolkit focused on identifying SNPs and INDELs in germline DNA and RNAseq data, with expanded capabilities to include somatic variant calling, copy number variation, and structural variation analysis.
FreeBayes	https://github.com/freebayes/freebayes	[74]	A Bayesian haplotype-based genetic polymorphism discovery and genotyping tool for variant calling.
Strelka2	https://github.com/Illumina/strelka	[75]	A variant caller capable of identifying germline and somatic small variants from DNA sequencing data.
Indelocator	NA	[77]	A somatic INDEL caller specifically designed for identifying insertions and deletions in DNA sequencing data.
MuSE	https://github.com/wwylab/MuSE	[78]	A somatic point mutation caller developed for detecting point mutations in tumor genomes.
MuTect	https://github.com/broadinstitute/mutect	[79]	A widely used somatic mutation caller.
Pindel	https://github.com/genome/pindel	[80]	A tool for detecting structural variants, such as large deletions, medium-sized insertions, inversions, tandem duplications, and other genomic rearrangements, from paired-end short reads.
RADIA	https://github.com/aradenbaugh/radia	[81]	A tool that integrates RNA and DNA sequencing data for somatic mutation detection.
SomaticSniper	https://github.com/genome/somatic-sniper	[82]	A tool designed for identifying somatic point mutations in WGS data.
VarScan	https://varscan.sourceforge.net/	[83]	A tool designed for somatic mutation and copy number alteration discovery in cancer using exome sequencing data.
RNASeq data analysis			
Cufflinks	http://cole-trapnell-lab.github.io/cufflinks/	[87]	A tool for transcriptome assembly and differential expression analysis of RNA-Seq.
StringTie	https://ccb.jhu.edu/software/stringtie/	[88]	A tool for transcript assembly and quantification from RNA-Seq data.
STAR-Fusion	https://github.com/STAR-Fusion/STAR-	[90]	A tool for identifying fusion transcripts from RNA-Seq data.

	Fusion/wiki		
Arriba	https://github.com/suhrig/arriba	[91]	A tool for gene fusion detection from RNA-Seq data.
STAR-SEQR	https://github.com/ExpressionAnalysis/STAR-SEQR	[90]	A tool for detecting and quantifying RNA Fusions from RNA-Seq data.
IRFinder	https://github.com/williamritchie/IRFinder	[93]	A tool dedicated to detecting intron retention events from RNA-Seq data
REdiscoverTE	http://research-pub.gene.com/REdiscoverTEpaper/	[94]	A computational method for quantifying genome-wide transposable element (TE) expression in RNA sequencing data.
CIRIquant	https://github.com/bioinfo-biols/CIRIquant	[96]	A tool for circRNA detection and quantification in RNA-Seq data.
Ribo-seq data analysis			
Ribotricer	https://github.com/smithlabcode/ribotricer	[101]	A tool designed for the detection of short and long active ORFs using Ribo-seq data
RiboHMM	https://github.com/rajanil/riboHMM	[102]	A tool that utilizes a mixture of hidden Markov models to infer translated sequences using Ribo-seq data.
RibORF	https://github.com/zhejilab/RibORF	[103]	A tool developed for Identifying genome-wide translated open reading frames using Ribo-seq data.
PRICE	https://github.com/erhard-lab/price	[104]	A tool that uses an EM algorithm to identify ORFs from Ribo-seq data
Ribo-TISH	https://bioinformatics.mdanderson.org/public-software/ribo-tish/	[105]	A tool focused on translation initiation site identification using Ribo-seq data.
Ribo-seq ORFs	https://www.gencodegenes.org/pages/riboseq_orfs/	[99]	A consensus set of Ribo-seq ORFs identified by seven experimental publications mapped to GENCODE version 35 annotations.
Customized database generation			
CustomizedProDB	https://bioconductor.org/packages/release/bioc/html/customProDB.html	[113]	A tool that enables the generation of customized protein database from DNA and RNA sequencing data.
JUMPg	https://github.com/liyuxin-bioinformatics/JUMPg	[114]	A proteogenomics software pipeline for customized database building, tag-based database search, peptide-spectrum match filtering, and data visualization.
PROTEOFORMER	https://github.com/Biobix/proteoformer	[115]	A proteogenomic pipeline that uses Ribo-seq data to generate a protein database.
pgdb	https://github.com/nf-core/pgdb	[116]	A workflow for generating ENSEMBL-based proteogenomics databases to boost the identification of non-canonical peptides.
DDA immunopeptidomic data analysis			
X!Tandem	https://www.thegpm.org/tandem/	[34]	One of the early open-source database search tools for peptide identification.
MaxQuant	https://www.maxquant.org/	[35]	A widely used software for peptide identification and quantification.
Comet	https://uwpr.github.io/Comet/	[32,107]	A database search tool with an extension for global amino acid variant and PTM analysis using the PSI extended FASTA format.
MS-GF+	http://proteomics.ucsd.edu/software-tools/ms-gf/	[33]	A sensitive and universal database search tool for peptide identification.
MSFragger	https://msfragger.nesvilab.org/	[37]	A fast database search tool based on fragment ion indexing.

Open-pFind	http://pfind.org/software/pFind/index.html	[38]	A sequence-tag-based open search engine for peptide identification.
PROMISE	https://github.com/merbllab/PROMISE	[11]	A protein modification integrated search engine for comprehensive peptide identification.
Neo-Fusion	https://github.com/zrolfs/MetaMorpheus/tree/Neo-Fusion	[123]	A tool for the discovery of spliced peptides in tandem MS data.
DIA immunopeptidomic data analysis			
OpenSWATH	http://openswath.org	[53]	A tool for the analysis of DIA data using the targeted extracted ion chromatogram (XIC) approach.
Spectronaut	https://biognosys.com/software/spectronaut/	[54]	A commercial software package designed for analyzing DIA data.
Skyline	https://skyline.ms/	[55]	An open-source software platform for targeted proteomics data analysis, supporting various workflows including DIA/SWATH.
DIA-NN	https://github.com/vdemichev/DiaNN	[56]	A universal automated software suite developed for DIA data analysis.
EncyclopeDIA	https://bitbucket.org/searleb/encyclopedia/wiki/Home	[57]	A library search engine designed for the analysis of DIA data.
MaxDIA	https://github.com/JurgenCox/compbio-base	[58]	A software platform integrated within the MaxQuant software environment, dedicated to the analysis of DIA data.
PEAKS	https://www.bioinfor.com/	[59]	A comprehensive proteomics tool that supports both DDA and DIA data analysis.
**PSM Rescoring to improve peptide identification**			
Percolator	https://github.com/percolator/percolator	[45]	A tool that uses semi-supervised learning for PSM rescoring to improve peptide identification from shotgun proteomics data.
MS-Rescue	http://www.cbs.dtu.dk/cgibin/sw_request?msrescue	[44]	A computational pipeline designed to increase the quality and yield of immunopeptidomics experiments.
MHCquant	https://github.com/nf-core/mhcquant	[46]	A pipeline specifically developed for identifying and quantifying MHC eluted peptides from mass spectrometry raw data.
DeepRescore	https://github.com/bzhanglab/DeepRescore	[41]	A tool that leverages deep learning techniques to improve peptide identification in immunopeptidomics studies.
**Deep learning-based retention time and fragment ion intensity prediction**			
Prosit	https://github.com/kusterlab/prosit	[48]	A deep learning architecture designed for predicting the chromatographic retention time and fragment ion intensity of peptides.
AutoRT	https://github.com/bzhanglab/AutoRT	[49]	A deep learning tool developed for predicting peptide retention time in chromatographic separations.
DeepMass	https://github.com/verilylifesciences/deepmass	[50]	A deep learning tool developed for predicting peptide fragment ion intensities.
pDeep	https://github.com/pFindStudio/pDeep	[51]	A deep learning tool developed for predicting peptide fragment ion intensities.
Quality control in noncanonical peptide identification			
NetMHCpan	https://services.heal	[125]	A tool that predicts the binding of peptides to any major

	thtech.dtu.dk/servic es/NetMHCpan-4.1/		histocompatibility complex (MHC) molecule of known sequence using artificial neural networks.
MHCflurry	https://github.com/o penvax/mhcflurry	[126]	A tool specifically designed for predicting peptide-MHC class I binding affinity.
HLAthena	http://hlathena.tools	[127]	A tool that provides allele- and length-specific as well as pan-allele-pan-length prediction models for endogenous peptide presentation.
PepQuery	http://www.pepquer y.org/	[128]	A universal targeted peptide search engine used for identifying or validating known and novel peptides of interest in any local or publicly available mass spectrometry-based proteomics datasets.
PDV	http://pdv.zhang-lab.org/	[129]	A proteomics data viewer that allows visualization of different types of proteomics data, including database searching results, de novo sequencing results, proteogenomics files, MS/MS raw data, and data from public proteomics databases.
Cancer-specificity determination			
TCGA data	https://portal.gdc.ca ncer.gov/	[130]	Comprehensive molecular characterization of over 20,000 primary cancer and matched normal samples spanning 33 cancer types.
CPTAC data	https://proteomic.da tacommons.cancer. gov/pdc/cptac-pancancer	[131, 132]	An ongoing effort to conduct a comprehensive and interconnected proteogenomic characterization of the most prevalent types of cancer.
GTEx data	https://gtexportal.or g/home/	[133]	A comprehensive public resource that enables the study of gene expression and regulation in 54 non-diseased tissue sites across nearly 1000 individuals.
Immunopeptidomics databases			
HLA Ligand Atlas	https://hla-ligand-atlas.org/	[134]	A comprehensive collection of tissue and HLA allele specific HLA ligands that are naturally presented. The data was generated through standardized mass spectrometry experiments and analyzed using a well-defined computational analysis workflow.
caAtlas	https://www.zhang-lab.org/caatlas/	[39]	A comprehensive resource for the selection and prioritization of peptides for immunogenicity testing and cancer immunotherapy development. It is built upon publicly available MS-based human immunopeptidomic datasets from 311 cancer samples covering nine cancer types and 707 non-cancerous samples.
Immunogeneicity prediction			
NetTepi	https://services.heal thtech.dtu.dk/servic es/NetTepi-1.0/	[137]	A tool for predicting T-cell epitopes from protein sequences.
PRIME	https://github.com/G fellerLab/PRIME	[138]	A predictor of immunogenic epitopes.
